# Advances in Clinical and Molecular Research of Biomaterials in Dentistry: The New Era for Dental Applications

**DOI:** 10.3390/jcm11154512

**Published:** 2022-08-02

**Authors:** Gaetano Isola, Teresa Lombardi

**Affiliations:** 1 Department of General Surgery and Surgical-Medical Specialties, School of Dentistry, University of Catania, 95124 Catania, Italy; 2 Department of Health Sciences, Magna Græcia University, 88100 Catanzaro, Italy

Biomaterials in dentistry play a fundamental role in the quality of regeneration mechanisms and in healing following different rehabilitation techniques [1]. The different fields of dentistry, including periodontology and oral and implant surgery are the main beneficiaries of the efficacy of biomaterials, which, from a success at a biomolecular level, translates into a clinical success highly interconnected with the surgical method practiced [2], hard and soft tissue healing [3] and surgical flaps used [4]. In recent decades, different approaches have been analyzed and developed in order to optimize the various dental biomaterials with the aim of better meeting the clinical and aesthetic needs of patients. In this regard, the efforts by the researchers focused on the stability and biological ability of the material, to improve its biocompatibility, speed of integration and implement the processes of osteoinduction and tissue regeneration [5]. Currently, the most frequently used dental materials include titanium alloy composites, bone substitutes and progenitor cells related to bone and periodontal tissues [5], with a clear role played by the study of the behavior of materials with tissue bioengineering analyses.

More specifically, the classification of biomaterials divides them into bioinert, active or biodegradable materials. Specifically, bio-inert materials, including titanium screws and plates, are those that do not interact with the host tissue/environment in which they are inserted, with the main objective of maintaining the fixation of a tissue in order to stimulate its regenerative or reparative processes. On the other hand, bioactive materials have the purpose of interacting directly with the surrounding environment, obtaining a bilateral mechanism of active interruption, inducing the release of biological substances or mediators useful for improving tissue healing capacity [6].

Therefore, in order to clinically choose the most appropriate material for treatment, it is of fundamental importance to know its structural and biological mechanisms, through the biomimetic processes of the material. In other words, the correct choice of a biomaterial in the dental field is the primary factor determining the patient’s medium- and long-term rehabilitation successes. In this regard, the understanding of the progress in dental biomaterials that has developed in recent years would lead to appropriate applications of the same in the specific type of rehabilitation, in order to comply with the aesthetic, functional and biological requirements of the case to be treated, and at the same time to correctly set the rehabilitation workflow and timing [7].

In fact, recently in dentistry, biological and tissue engineering techniques have dramatically improved the quality, quantity and speed of regeneration of hard and soft tissues with an ever-greater effect of inducing tissue regeneration at the expense of classic repair mechanisms, with an implementation clear of the quality of dental rehabilitation [8,9]. However, there are still several gaps that allow an optimal understanding of the functioning of the biomaterial in tissue induction from progenitor mesenchymal cells to mature target tissue, especially for tissues such as dentin and periodontal ligament; in this regard, more and more efforts are made to try to regenerate dentinal, enamel, periodontal, nerve and pulp cells when they are lost due to illness or incorrect rehabilitation. The use of dental stem cells as primary and essential cellular sources to facilitate tissue regeneration in dentistry has now been properly introduced [10]. However, because the cellular mechanisms underlying successful new bone formation are not well understood, there is a need for a further explanation of the cellular mechanisms underlying the formation of hard and soft tissues in dental clinical practice.

Among those, in periodontology, over the years, various evidence has reported the success of periodontal regeneration obtained through the use of regenerative strategies to restore the lost periodontal root and gingival tissue in order to be able to use them in periodontal surgery techniques and, recently, non-surgical debridement [11]. However, these regenerative therapeutic approaches, without the use of supporting osteoconductive materials, have been shown to have some limitations in inducing true periodontal regeneration with the formation of new tissue. Specifically, through investigation methods with the use of tissue engineering, various methods of periodontal regeneration were studied with the combination of bone grafts, growth factors, 3D bone scaffolds and amelogenins, to be used in regenerative periodontal surgery with and without the use of membranes [12]. To this end, both guided tissue regeneration (GTR) and guided bone regeneration (GBR) [13] have been introduced to properly support tissue healing mechanisms with or without the use of biomaterials. However, the new approaches of tissue engineering in periodontology have shown that for a correct and full tissue regeneration of soft and hard tissues, regenerative biomaterials are needed that allow us to obtain key elements for regeneration including progenitor cells, scaffolds or associated support matrices and tissue signaling molecules [14,15]. Biomaterials made with bioengineering techniques have shown that they can act both as a scaffold, with osteoconductive activity that supports at the same time a multiphase and bioactive mechanism of osteogenesis and soft tissue regeneration that allow us to obtain an excellent periodontal regeneration, with clinical and mind-blowing biologicals [16]. More specifically, even natural biomaterials, with amelogenins and growth factors used in liquid or 3D printed through a highly personalized and customized workflow, are highly used in periodontology thanks to their cellular affinity and biocompatibility capabilities, with low tissue toxicity or effects of rejection related to inflammatory responses or immune reactions by the host [17].

In implantology, since bone healing is fundamental in the bone–biomaterial interaction, the surface feature is important in accelerating osseointegration. In this field, many studies have focused on improving the condition of the Ti surface by incorporating optimal surface roughness, such as machined sandblasted, acid-etched, anodized, laser modifications and surface coating with osteoconductive biomaterials. Furthermore, hydroxyapatite, calcium phosphate and biomolecules (BMP-2) have been developed to improve osseointegration [18]. In this regard, osteoinductive or conductive biomolecules have been shown to be essential in the main and early differentiation mechanisms of mesenteric cells into mature and functional bone cells, accelerating the mechanisms of osteogenesis and osseointegration. Specifically, the mechanisms of osteoinductivity and conductivity have been demonstrated when the biomaterial used has the ability to mimic as much as possible the extracellular matrix (ECM) and biomimicry of bone tissue, an essential factor in the success of implantology and dentistry [19,20].

Based on these considerations, new biomaterials and technologies play a key role in modern dentistry’s development, which requires high-quality multidisciplinary research. Understanding recent advances in the biomaterial of dentistry would lead to finding the best application and most successful treatment strategies for improving patient treatment outcomes.

Whilst several obstacles to successful clinical translation have been highlighted in this review, none are beyond resolution [21,22,23,24]. The ultimate aim should be to assist in the decision making of researchers when they may be initiating novel advances in oral-related therapies, by ensuring that they are cognizant of past errors and limitations, whilst at the same time recognizing present hurdles. Clinicians should also be alert to the complexities affecting the arrival in their surgeries of new products and technologies.

Despite these challenging features, even more, continued efforts are making significant progress in developing bioactive scaffolds with the potential to regenerate periodontal tissues. Active interdisciplinary collaborations between biomaterial scientists, biologists, and chemical and clinical engineers will serve as a key catalyst, potentially leading us towards the functional regeneration of periodontal tissues.

Biomaterial science is a crucial element in the advance of all clinical disciplines. In this regard, a strict collaboration must be encouraged between clinicians and scientists that may yield discussions which will cultivate innovative strategies for improved and enhanced patient care.

## Data Availability

Data are available from the corresponding author upon reasonable request.
